# Impact of intrathecal morphine analgesia on the incidence of pulmonary complications after cardiac surgery: a single center propensity-matched cohort study

**DOI:** 10.1186/s12871-017-0398-z

**Published:** 2017-08-22

**Authors:** Christoph Ellenberger, Tornike Sologashvili, Krishnan Bhaskaran, Marc Licker

**Affiliations:** 1 Department of Anesthesiology, Pharmacology and Intensive Care, University Hospital of Geneva, Rue Gabrielle-Perret-Gentil 4, -1211 Geneva, CH Switzerland; 2 Division of Cardiovascular Surgery, University Hospital of Geneva, rue Gabrielle-Perret Gentil, Geneva, 1211 Switzerland; 3 London School of Hygiene and Tropical Medicine, London, UK

**Keywords:** Spinal analgesia, Postoperative respiratory failure, Opiates, Pulmonary complications

## Abstract

**Background:**

Acute pain and systemic opioids may both negatively impact respiratory function after cardiac surgery. This study analyzes the local practice of using intrathecal morphine analgesia (ITMA) with minimal parenteral opioid administration in cardiac surgery, specifically the impact on postoperative pulmonary complications (PPCs).

**Methods:**

Data from adult patients who underwent elective cardiac surgery between January 2002, and December 2013 in a single center were analyzed. Propensity scores estimating the likelihood of receiving ITMA were used to match (1:1) patients with ITMA and patients with intravenous analgesia (IVA). Primary outcome was PPCs, a composite endpoint including pneumonia, adult respiratory distress syndrome, and any type of acute respiratory failure. Secondary outcomes were in-hospital mortality, cardiovascular complications, and length of stay in the intensive care unit (ICU) and hospital.

**Results:**

From a total of 1′543 patients, 920 were treated with ITMA and 623 with IVA. No adverse event consequent to the spinal puncture was reported. Propensity score matching created 557 balanced pairs. The occurrence of PPCs in patients with ITMA was 8.1% vs. 12.8% in patients with IVA (odds ratio, 0.6; 95% CI, 0.40–0.89; *p* = 0.012). Fewer patients with ITMA had a prolonged stay in the ICU (> 4 days; 16.5% vs. 21.2%, *p* = 0.047) or in the hospital (> 15 days; 25.5% vs. 31.8%. *p* = 0.024). In-hospital mortality and cardiovascular complications did not differ significantly between the two groups.

**Conclusion:**

In this study involving cardiac surgical patients, ITMA was safely applied and was associated with fewer PPCs.

**Electronic supplementary material:**

The online version of this article (doi:10.1186/s12871-017-0398-z) contains supplementary material, which is available to authorized users.

## Background

Postoperative pulmonary complications (PPCs) such as pneumonias and acute respiratory failure are important contributors to major morbidity and mortality, prolonged hospital stay and reduced long-term survival following major surgical procedures [1]. Patients undergoing open heart surgery are at high risk of PPCs [2–4]. In the immediate postoperative period, vital capacity and functional residual capacity are decreased by approximately 30 to 60% and progressively recover over the next 10 to 15 days [5, 6]. This reduction in pulmonary volumes is strongly related to the degree of pain that prevents the patient to cough adequately and to take deep breaths in order to clear the airways and to reopen collapsed alveolar areas [7–9].

Currently, pain management in cardiac surgery is largely based on systemic opioids, combined with nonsteroidal anti-inflammatory drugs and paracetamol. However, opiate-based analgesia produces dose-related adverse events such as respiratory depression, nausea/vomiting, constipation, pruritus and mental confusion, which all may impair functional recovery and delay patient discharge from the hospital. [10] In addition, opioid-induced hyperalgesia may occur when large intravenous doses are given intraoperatively [11].

A possible way to provide efficient analgesia with minimal systemic opioid administration is the intrathecal administration of a small dose of morphine (“spinal analgesia”) [12, 13]. Analgesia starting 30 min and lasting up to 24 h has been reported after single-shot injection as a result of direct activation of the μ opioid receptors in the substantia gelatinosa of the posterior spinal cord, with no interference with blood pressure and heart rate control. [12]

To facilitate postoperative awakening and extubation while improving postoperative pain control, intrathecal morphine analgesia (ITMA) with minimal systemic opioid administration has been implemented in cardiac surgery at our institution since 2002. In the current study, we have questioned the safety and the clinical impact of ITMA in our local cohort of cardiac surgical patients. Therefore, based on administrative and clinical hospital data, we have analyzed the relationship between the type of intraoperative analgesic regimen and the occurrence of major adverse events, particularly the incidence of PPCs.

## Methods

### Patient population and data collection

The study was approved by the local ethic committee (CER file number: 14-080R) with a waiver of the requirement for obtaining written informed consent. We collected data concerning all patients undergoing elective cardiac surgery between January 1, 2002 and December 31, 2013. The patients were identified in the hospital electronic charting system by searching for the *Swiss classification of surgical interventions* (CHOP) code for cardiopulmonary bypass (CPB). Emergent cases, patients hospitalized in the intensive care unit (ICU) before surgery, and those scheduled for implantation of a ventricular assist device were excluded from the study. Also patients requiring redo surgery (e.g., for bleeding or cardiac tamponade) were excluded from the analysis, because spinal analgesia was not repeated and all patients treated with intravenous opioids. If a patient underwent more than one cardiac surgery during the study period, only the first procedure was included in the analysis.

A data set was created containing all World Health Organization International Classification of Diseases codes (ICD-10), and all CHOP codes related to the patient’s hospital stay, as well as demographic data (age, sex), length of hospital stay, length of stay in the ICU, and the date of death. Information regarding the type of the analgesia technique (ITMA or standard intravenous analgesia (IVA)) was collected by scanning anesthesia worksheets (from January 1st, 2002 to December 31, 2006) or from the electronic anesthesia records (from January 1st, 2007 to December 31, 2013). ICD-10 codes were used for classification of the co-morbidities based on the methodology of Quan et al. [14] (Additional file 1: Annex A). The type of surgery was stratified into 4 categories based on the CHOP codes: coronary artery bypass grafting, valve surgery, combined surgery, and other surgery (e.g. surgery of the ascending aorta or adult congenital heart surgery).

Postoperative ICU data regarding the duration of mechanical ventilation, the need for non-invasive ventilation (NIV) and/or continuous positive airway pressure (CPAP) therapy, as well as the daily administration of morphine and fentanyl and pain scores were extracted from the electronic ICU records. Due to the launching date of the advanced information system for the ICU (Clinisoft) only data from patients operated after 2006 were available.

### Perioperative analgesia

ITMA was proposed to all patients in the absence of hemostatic disorders, abnormal coagulation tests, or antiplatelet therapy other than aspirin. If a patient accepted ITMA, intraoperative analgesia consisted in an intrathecal single-shot injection of 10 mcg/kg morphine in the lumbar space (L3-L4) using a 25-gauge spinal needle prior to induction of general anesthesia, whereas systemic administration of opioids was limited to the time of anesthesia induction, skin incision and sternotomy (sufentanyl 15–25 mcg or fentanyl 150–250 mcg). In the IVA group (patients refusing ITMA or patients with a contraindication for ITMA), intravenous opiates were administered as a bolus during anesthesia induction (sufentanyl 10–20 mcg or fentanyl 100–200 mcg) and before skin incision sufentanyl 10–20 mcg or fentanyl 100–200 mcg), and continuously thereafter until skin closure (sufentanyl 10–25 mcg/h or fentanyl 100–250 mcg/h).

### Perioperative management

Standard monitoring included an arterial line, a central venous catheter, transesophageal echocardiography (TEE) and bispectral analysis of the electroencephalographic signals (BIS). After anesthesia induction and orotracheal intubation, patients were mechanically ventilated using tidal volumes of 6–8 ml.kg^−1^ of predicted body weight, a positive end-expiratory pressure of 5–10 cm of water and regular recruitment maneuvers (at least once an hour). Anesthesia was maintained with propofol to target BIS values 40–60. To enhance myocardial protection (preconditioning) sevoflurane 1–1.5 MAC was administrated for at least 30 min in the prebypass period [15].

Cardiac surgery was performed via full sternotomy. The circuit of the nonpulsatile CBP machine was primed with 1 L of crystalloids and 1 L of hydroxyethyl starch 6% 130/0.4. Myocardial protection was achieved by antegrade infusion of hyperkaliemic cold blood. Following aortic cross-clamping, the lungs were disconnected from the ventilator. All patients received an intravenous bolus of 15 mg·kg^−1^ tranexamic acid with an additional dose of 10 mg·kg^−1^ in the CPB priming fluid. Weaning from the CPB was guided by TEE assessment and invasive pressure monitoring [16]. Besides titration of fluids, cardiovascular medications were given to target specific hemodynamic endpoints: LV end-diastolic diameter (up to preoperative values, respectively 2.2 to 2.8 cm/m^2^), mean arterial pressure (MAP) between 65 and 90 mmHg and HR between 70 and 100 beats/min.

### Study endpoints

The primary outcome was PPCs, a composite endpoint of the occurrence of pneumonia (with the exception of viral pneumonia), adult respiratory distress syndrome (ARDS) and any other cause of acute respiratory failure during hospitalization. ICD-10 codes were used to retrieve pneumonia, ARDS and acute respiratory failure (Additional file 1: Annex A). Postoperative atelectasis was not included in the primary outcome as its identification could be dependent on subjective evaluation by the clinician in charge of the patients. Secondary outcomes were in-hospital mortality at 60 days, cardiovascular adverse events including acute myocardial infarction, stroke, and the need for intra-aortic balloon pump as a sign of postoperative heart failure, as well as the length of stay in the ICU and hospital. We further searched the database for any neurological complication related to ITMA.

In patients operated later than 2006 we analyzed the duration of mechanical ventilation and the need for NIV and CPAP in the two groups. We also compared the number of patients receiving morphine and fentanyl parenterally, the total dose administered, and the postoperative pain scores within the first two days after surgery.

### Analysis

The computer software Stata 14 (Stata Corp, College Station, TX, USA) was used for statistical analysis. Propensity score matching was used to reduce confounding, respectively to adjust for baseline differences between the two groups. A non-parsimonious logistic regression model was constructed estimating the likelihood that any given individual in the cohort would be in the ITMA group, given the set of baseline variables. The following variables were included in the model: age, gender, type of surgery, as well as the following comorbidities: diabetes, high blood pressure, hypercholesterolemia, coronary heart disease, peripheral vascular disease, chronic pulmonary disease, smoking, obesity, and chronic kidney disease. The functional form of the regression equation was assessed with the Hosmer-Lemeshow test showing a good fit (*p* = 0.394), therefore no interaction terms were included in the model.

Patients with ITMA were then individually matched (1:1) with patients with IVA based on the similarity of the propensity score [17]. A nearest neighbor matching technique without replacement was carried out using the Stata ‘psmatch2’ module [18]. Caliper distance was set at 0.1 standard deviations of the sample estimated propensity scores because larger calipers (0.20 or 0.25 standard deviations) were unable to balance the cohorts [17]. The ability of the model to balance the cohorts was assessed using standardized differences. Differences of absolute value less than 10% were considered to indicate well balanced covariates [19]. Because ICU data were only available in patients operated later than 2006, we stratified the analysis into two periods (2002–2006 and 2007–2013) to avoid the creation of matched pairs with missing data. Conditional logistic regression was used to compare categorical outcomes in the matched dataset, Wilcoxon signed-rank tests were used to compare quantitative outcomes.

### Sensitivity analyses

Overall patient management may have changed/improved over the study period. To evaluate this potential source of confounding, a post hoc sensitivity analysis was carried out to assess the effect of ITMA on PPCs after balancing the year of surgery in the two groups. The presence of hemostatic disorders could represent another source of confounding, since these disorders are contraindications for ITMA and are potentially associated with worse outcome due to increased postoperative bleeding. Because it was not possible to identify hemostatic disorders reliably in the electronic charting system, an estimator of postoperative bleeding was created, based on the codes for post-procedural hemorrhage, acute post-hemorrhagic anemia, and hypovolemic shock. A second *post-hoc* sensitivity analysis was then carried out to assess the effect of ITMA on PPCs after balancing postoperative bleeding in the two groups. For the sensitivity analyses the same statistical model was used, with the exception that the year of surgery, respectively postoperative bleeding, was included.

## Results

**Fig. 1:**
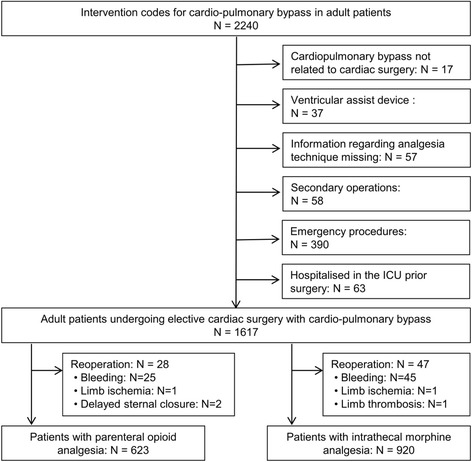
Patient enrolment

Patient enrolment is shown in Fig. 1. Overall, 1′543 patients were analyzed, 920 (59.6%) with ITMA and 623 (40.4%) with IVA. Baseline characteristics are shown in Table 1. Before matching, patients in the IVA group were slightly older and more likely to have risk factors for coronary heart disease including diabetes, dyslipidemia, smoking, and hypertension. They also underwent more often coronary artery bypass graft (CABG) surgery. The algorithm provided 557 pairs of ITMA/IVA patients. The matching process achieved a good balance for all comorbidities and the type of surgery.

**Table 1:** Patient characteristics before and after propensity score matching

Patient characteristics	Patients with IVA		Patients with ITMA		Standardized difference	Patients with IVA		Patients with ITMA		Standardized difference
	*N* = 623		*N* = 920		%	*N* = 557		*N* = 557		%
Age (years) ^a^	65.1	(15.3)	61.4	(17.2)	−22.8	64.7	(15.7)	63.8	(16.0)	−5.1
Sex (male)	424	(68.1)	610	(66.3)	3.7	372	(66.8)	381	(68.4)	3.4
Hypertension	344	(55.2)	432	(47.0)	−16.6	295	(52.3)	288	(51.7)	−2.5
Diabetes	134	(21.5)	159	(17.3)	−10.7	112	(20.1)	113	(20.3)	0.5
Hypercholesterolemia	137	(22.0)	180	(19.6)	−6.0	116	(20.8)	125	(22.4)	4.0
Coronary heart disease	331	(53.1)	335	(36.4)	−34.1	269	(48.3)	263	(47.2)	−2.2
Peripheral vascular disease	127	(20.4)	171	(18.6)	−4.5	108	(19.4)	120	(21.5)	5.4
Chronic pulmonary disease	63	(10.1)	77	(8.4)	−6.0	46	(8.3)	48	(8.6)	1.2
Smoking	102	(16.4)	87	(9.5)	−20.7	65	(11.7)	62	(11.1)	−1.6
Obesity	57	(9.2)	82	(8.9)	−0.8	51	(9.2)	56	(10.1)	3.1
Chronic kidney disease	79	(12.7)	79	(8.6)	−13.3	63	(11.3)	55	(9.9)	−4.7
Parsonnet score	14.9	(9.0)	13.7	(8.7)	−13.4	14.7	(9.0)	14.7	(9.1)	0.5
Type of surgery										
CABG	187	(30.0)	182	(19.8)	−23.8	151	(27.1)	136	(24.4)	−6.3
Valve surgery	291	(46.7)	530	(57.6)	21.9	281	(50.5)	283	(50.8)	0.7
Combined surgery	110	(17.7)	123	(13.4)	−11.9	91	(16.3)	94	(16.9)	1.5
Other	35	(5.6)	85	(9.2)	13.8	34	(6.1)	44	(7.9)	6.9

Data given as number (percentage) unless otherwise indicated

*IVA* intravenous analgesia; *ITMA* intrathecal morphine analgesia; *CABG* coronary artery bypass graft

^a^ Data given as mean (standard deviation)

The primary and secondary study outcomes are summarized in Table 2. In the whole cohort of 1′543 patients, PPCs occurred in 167 patients (9.0% in the ITMA group vs. 13.5% in the IVA group, *p* = 0.006), and 63 patients died within the first 60 days after surgery (3.2% in the ITMA group vs. 5.5% in the IVA group, *p* = 0.025). In the propensity matched cohort, the proportion of patients with PPCs in patients with ITMA was 8.1% compared to 12.8% in patients with IVA (odds ratio, 0.6; 95% CI, 0.40–0.89; *p* = 0.012). All three clinical outcomes of the composite endpoint occurred less frequently in the ITMA group. Overall, pneumonia and acute respiratory failure were the most frequent adverse events.

**Table 2:** Patient outcomes before and after propensity score matching

	Entire sample				Propensity Matched Cohort					
Outcomes	IVA		ITMA		IVA		ITMA		OR (95% CI)	*P-*Value
	*N* = 623		*N* = 920		*N* = 557		*N* = 557			
Respiratory complications	84	(13.5)	83	(9.0)	71	(12.8)	45	(8.1)	0.60 (0.40–0.89)	0.012
Acute respiratory insufficiency	49	(7.9)	47	(5.1)	45	(8.1)	22	(4.0)	0.45 (0.21–0.98)	0.047
Pneumonia	52	(8.4)	43	(4.7)	43	(7.7)	27	(4.9)	0.60 (0.36–0.99)	0.048
ARDS	5	(0.8)	3	(0.3)	5	(0.8)	0	(0.0)	NA	NA
*Secondary outcomes*										
Death (60d post-op)	34	(5.5)	29	(3.2)	33	(5.9)	22	(4.0)	0.66 (0.38–1.14)	0.134
Myocardial infarction	61	(9.8)	48	(5.2)	49	(8.8)	33	(5.9)	0.66 (0.42–1.04)	0.072
Stroke	25	(4.0)	31	(3.7)	20	(3.6)	24	(4.3)	1.22 (0.66–2.28)	0.528
IABP	18	(2.9)	29	(3.2)	18	(2.9)	19	(3.4)	1.06 (0.54–2.10)	0.862
Postoperative Bleeding ^a^	63	(10.1)	66	(7.2)	56	(10.1)	41	(7.4)	0.71 (0.46–1.08)	0.111
ICU stay, days ^b^	2	(1–4)	2	(1–3)	2	(1–4)	2	(1–3)		0.014 ^c^
ICU stay >4 days	135	(21.7)	146	(15.9)	118	(21.2)	92	(16.5)	0.73 (0.54–0.99)	0.047
Hospital stay days ^b^	13	(10–17)	12	(9–16)	13	(10–17)	12	(9–16)		0.038 ^c^
Hospital stay >15 days	197	(31.6)	247	(26.9)	177	(31.8)	142	(25.5)	0.74 (0.57–0.96)	0.024

Data given as number (percentage) unless otherwise indicated. Conditional logistic regression was used for statistical tests in the matched cohort unless otherwise indicated

*IVA* intravenous analgesia; *ITMA* intrathecal morphine analgesia; *ARDS* adult respiratory distress syndrome; *IABP* intra-aortic balloon pump; *ICU* intensive care unit

^a^ Composite of post-procedural hemorrhage, acute post-hemorrhagic anemia, and hypovolemic choc

^b^ Data given as median (interquartile range)

^c^ Wilcoxon signed-rank test

The proportion of patients with prolonged ICU stay (longer than 4 days) was significantly lower in patients with ITMA (16.5% vs. 21.2%; OR 0.73, 95%CI 0.54–0.99, *p* = 0.047). Likewise, the proportion of patients staying longer than 15 days in the hospital was also lower in the ITMA group (26.9% vs. 31.6%; OR 0.74, 95%CI 0.57–0.96, *p* = 0.024). Mortality at 60 days, the need for intra-aortic balloon pump and the occurrence of stroke did not differ significantly between the two groups. There were no neurological complications or spinal hematomas related to the spinal puncture in patients with ITMA.

The ICU data of patients operated later than 2006 are shown in Table 3. The duration of mechanical ventilation as well as the administration of CPAP and NIV were similar in both groups. During the first 24 h, the median pain score (on a scale of 0 to 10) was significantly lower in the ITMA group (1.8 [0.0–3.2] vs. 3.0 [1.3–4.0, *p* < 0.001) and fewer patients experienced a pain score ≥ 5 (7.2% vs. 15.1%; OR 0.45, 95%CI 0.26–0.78, *p* = 0.004). Fewer patients in the ITMA group were treated with morphine during the first 24 h (61.0% vs. 74.6%; OR 0.55 95%CI 0.40–0.76, *p* < 0.001) and the median dose per treated patient was significantly lower (8 mg [4–14] vs. 15 mg [8–24], *p* < 0.001). Likewise, fewer patients in the ITMA group were treated with fentanyl (26.6% vs 33%, OR 0.69, 95%CI 0.51–1.00, *p* = 0.052) with a median dose that was significantly lower (150 mcg [73–390] vs. 276 mcg [125–601], *p* = 0.002).

**Table 3:** ICU characteristics before and after propensity score matching

	Entire sample				Propensity Matched Cohort					
Outcomes	IVA		ITMA		IVA		ITMA		OR (95% CI)	*P-*Value
	*N* = 355		*N* = 648		*N* = 346		*N* = 346			
Respiratory treatment										
Mechanical ventilation, min ^a^	505	(275–1001)	458	(206–958)	501	(275–994)	496	(212–997)		0.790 ^b^
CPAP	267	(75.2)	483	(74.5)	260	(75.1)	259	(74.9)	0.99 (0.70–1.38)	0.931
min per patient treated ^a^	125	(75–215)	120	(60–240)	120	(72.5–213)	120	(75–240)		0.385 ^b^
NIV	81	(22.8)	153	(23.6)	79	(22.8)	75	(21.7)	0.94 (0.66–1.33)	0.722
min per patient treated ^a^	120	(60–240)	110	(60–220)	120	(60–240)	155	(60–275)		0.470 ^b^
Opioid administration										
*Day 1*										
Morphine	265	(74.7)	400	(61.7)	258	(74.6)	211	(61.0)	0.55 (0.40–0.76)	<0.001
mg per patient ^a^	15	(8–24)	8.5	(4–15.5)	15	(8–24)	8.0	(4–16)		<0.001 ^b^
Fentanyl	119	(33.5)	165	(26.1)	115	(33.2)	92	(26.6)	0.69 (0.51–1.00)	0.052
mcg per patient ^a^	276	(125–583)	160	(75–400)	276	(125–601)	150	(73–390)		0.002 ^b^
*Day 2*										
Morphine	127	(39.7)	196	(35.1)	123	(39.6)	111	(35.9)	0.86 (0.62–1.17)	0.334
mg per patient ^a^	8.0	(3–13)	8.0	(3–14)	7.0	(3–14)	8.0	(3–13)		0.275 ^b^
Fentanyl	50	(15.6)	86	(15.4)	48	(15.4)	50	(16.2)	1.05 (0.69–1.59)	0.831
mcg per patient ^a^	348	(100–1136)	175	(50–641)	348	(100–1170)	188	(100–545)		0.500 ^b^
Postoperative pain										
*Day1*										
Pain score ^a^	2.9	(1.3–4.0)	1.9	(0.0–3.3)	3.0	(1.3–4.0)	1.8	(0.0–3.2)		<0.001 ^b^
Pain score ≥ 5	51	(15.0)	50	(8.1)	50	(15.1)	24	(7.2)	0.45 (0.26–0.78)	0.004
*Day2*										
Pain score ^a^	2.4	(1.0–4.0)	2.0	(0.4–3.7)	2.5	(1.0–4.0)	2.0	(0.1–3.8)		0.853 ^b^
Pain score ≥ 5	40	(17.1)	42	(10.7)	39	(17.3)	2753	(12.1)	0.75 (0.38–1.46)	0.400

Data given as number (percentage) unless otherwise indicated. Conditional logistic regression was used for statistical tests in the matched cohort unless otherwise indicated

*IVA* intravenous analgesia; *ITMA* intrathecal morphine analgesia; *CPAP* continuous positive airway pressure; *NIV* non-invasive ventilation

^a^ Data given as median (interquartile range)

^b^ Wilcoxon signed-rank test

### Sensitivity analyses

The first sensitivity analysis was carried out to assess potential confounding related to changes in patient management over time. The propensity score–matching algorithm created 514 patient pairs who were well balanced for all baseline characteristics, including the year of surgery. The effect on the association between analgesia regimen and PPC was minimal and the occurrence of PPCs in patients with ITMA remained significantly lower compared with the IVA (odds ratio, 0.65; 95% CI, 0.44–0.95; *p* = 0.027).

The second sensitivity analysis was carried out to assess potential confounding related to the bleeding risk. The propensity score–matching algorithm created 561 patient pairs who were well balanced for all baseline characteristics as well as postoperative bleeding. Balancing postoperative bleeding in the two groups had only a small effect, and ITMA remained associated with lesser risk of PPCs (odds ratio, 0.66; 95% CI, 0.49–0.96; *p* = 0.030).

## Discussion

In this single center cohort study, the use of ITMA in elective cardiac surgery, together with restrictive parenteral opioid administration, was associated with a lower incidence of PPCs as defined by the occurrence of pneumonia, ARDS and other forms of acute respiratory failure during hospitalization. In addition, ITMA was also associated with lesser requirements of opioids and lower pain during the first postoperative day whereas fewer patients requiring a prolonged ICU and hospital stay.

Acute postoperative pain is a common problem after cardiac surgery [20]. Impaired coughing, shallow breathing, immobility and bed rest may lead to atelectasis and reduced clearance of pulmonary secretions increasing the risk of atelectasis, pneumonia and respiratory insufficiency [7, 21]. Deep breathing exercises have been shown effective in reducing atelectatic areas and improving pulmonary function in patients undergoing CABG surgery [22]. Adequate pain control is therefore essential and systemic opioids remain the cornerstone of postoperative analgesia after cardiac surgery. Unfortunately, the use of opioids itself is associated with dose-dependent adverse drug events including respiratory depression, dizziness/cognitive dysfunction, nausea/vomiting, constipation, and pruritus [23]. In particular older and sicker patients are at greater risk for opioid related adverse events. Therefore, the benefit of parenteral opioids in this population should be balanced against the risk of impaired functional recovery and delayed patient discharge from the hospital. [10]

Central neuraxial analgesia techniques can improve postoperative pain while reducing the need for systemic opioids. Conceptually, thoracic epidural analgesia is the most efficient analgesia technique and has been associated with a reduction in the incidence of PPCs after CABG surgery [24]. However, many anesthesiologists are reluctant to use epidural analgesia in cardiac surgery. The use of the heart lung machine during cardiac surgery mandates high levels of blood anticoagulation increasing the potential risk of puncture related epidural hematoma/neural trauma [25]. Therefore, ITMA may be an attractive alternative given its simplicity, high success rate, and a low risk profile. For these reasons, our cardiothoracic anesthesia team has adopted this analgesia technique in the routine perioperative care of cardiac surgical patients.

To our knowledge, this is the first study assessing the association between ITMA and PPCs in cardiac surgery. Previous studies have analyzed the effect of ITMA on postoperative lung function. In 2 randomized controlled trials, ITMA was associated with an improved forced vital capacity (FVC) and forced expiratory volume (FEV) after cardiac surgery [26, 27]. In contrast, dos Santos et al. found a similar decline of the FVC and FEV in patients with ITMA and IVA that was likely attributed to the high intravenous opiate doses that were given in both groups [28]. In a cohort of 595 patients undergoing major vascular surgery, we previously reported that ITMA was associated with a reduced risk of postoperative morbidity, particularly PPCs and renal dysfunction [29].

In contrast to most randomized trials, ITMA was not associated with a shorter duration of postoperative mechanical ventilation in our patient cohort. [27, 30–34] A plausible explanation may be the relatively high dose of intrathecal morphine administered. However, Shroff et al. found a shorter extubation time when using 10 mcg/kg of intrathecal morphine [34]. In most studies where ITMA facilitated early extubation, the intrathecal dose of morphine ranged from 4 to 8 mcg. It has been recently shown that, in minimal invasive cardiac surgery, 1.5 mcg/kg intrathecal morphine is sufficient to provide pain control. [35] Consistent with other studies, our findings confirmed the superior analgesia of intrathecal morphine that was associated with lower opioid requirements in cardiac surgical patients [20].

Two meta-analyses had been conducted to assess the association between ITMA and postoperative mortality respectively myocardial infarction. The first by Liu et al. included 668 patients from 17 randomized trials [24], the second by Zangrillo et al. included 1106 patients from 25 randomized trials [36]. Similar to our study, the authors found no significant association between ITMA and postoperative mortality (0.3 vs. 0.6%, respectively 0.4 vs. 0.4%) or myocardial infarction (3.9 vs. 5.7%%, respectively 2.1 vs. 2.7%).

Important limitations need to be discussed when interpreting the results of this study. First, this is an observational study of administrative data using propensity scores. Propensity scores can only balance for measured covariates. Therefore, the good balance of covariates in our matched groups does not indicate balance of unmeasured variables like preoperative medical treatment, laboratory values, blood transfusion, duration of cardio-pulmonary bypass/aortic cross-clamping or ventilator parameters. Furthermore, lung function tests after cardiac surgery to detect relevant changes in pulmonary function were not performed on a routine basis. However, most intraoperative variables are directly related to patient characteristics and the type of surgery which were well matched in the two groups. Also, anesthesia care was highly standardized and protective lung ventilation strategies were used in all patients. Still, the possibility that unmeasured variables (e.g., contraindications for ITMA such as hemostatic disorders) could bias the effect estimates of ITMA on PPCs needs to be considered. Conversely, the proportion of postoperative bleeding was similar in both groups and balancing for postoperative bleeding had no effect on the association between ITMA and PPCs. Anticoagulation and antiplatelet therapy (with the exception of aspirin) were in general interrupted three to seven days before cardiac surgery to avoid excessive bleeding.

Second, administrative databases may result in underreporting of comorbidities and outcome data [37]. However, this is difficult to assess because clear definition criteria for PPCs are lacking and the reported occurrence in the literature is extremely variable ranging from 8% to 79% [2]. The administrative codes also did not allow precise determination a major adverse event. For instance, patients with coronary artery disease may first present at the hospital with signs of a myocardial infarction (and getting a code for acute myocardial infarction) and then undergo uneventful elective CABG surgery. We found that 17.1% of the patients undergoing CABG had a code for acute myocardial infarction compared to 2.1% of the patients undergoing non-CABG surgery, indicating that the coding may overestimate the occurrence of postoperative myocardial infarction in these patients. Furthermore, adverse events occurring after the patient’s discharge would have been missed. Re-hospitalization would have generated a new hospital stay in the hospital electronic charting system. All ICD-10 codes for the adverse event would have been recorded in the new hospital stay.

Third, the study spanned over one decade and the practice of perioperative medicine and the profile of the cardiac surgery population may have changed over time. However, there was no significant variation over the years in the occurrence of PPC in our cohort and the sensitivity analysis adjusting for the year of surgery had no effect on the association between ITMA and PPC. Furthermore, the surgical and the anesthesia teams did not change, cardiopulmonary bypass techniques were also similar; the respiratory protocol to wean the patient from the ventilator was unchanged, efforts being made to extubate the patient as soon as physiological criteria were met. Finally, this is a single center study and the generalizability of the result may be limited. ITMA was introduced as part of a perioperative care protocol together with minimal systemic opioid administration. The ICU staff was aware of the chosen analgesia technique and could be influenced regarding treatment decisions.

## Conclusion

The results of this study suggest that improving postoperative pain control with a small dose of spinal morphine while minimizing systemic opioid administration may reduce the incidence of PPCs in patients undergoing elective cardiac surgery. These findings warrant a multicenter randomized trial to demonstrate the effectiveness of this simple and safe intervention, particularly in patients at high-risk of PPCs.

## Additional file

Additional file 1: ICD-10 codes used to define comorbidities and outcomes and CHOP codes used to define surgical procedures. (DOCX 14 kb)
