# Supplementary material for: Impact of intrathecal morphine analgesia on the incidence of pulmonary complications after cardiac surgery: a single center propensity-matched cohort study
Source: BMC Anesthesiol. 2017 Aug 22;17:109. doi: 10.1186/s12871-017-0398-z (PMC5567923; doi:10.1186/s12871-017-0398-z)
Supplement: Additional file 1: — ICD-10 codes used to define comorbidities and outcomes and CHOP codes used to define surgical procedures. (DOCX 14 kb) [file 12871_2017_398_MOESM1_ESM.docx]

**Annex A**

ICD-10 codes used to define comorbidities and outcomes:

Hypertension I10.x, I11.x-I13.x, I15.x

Diabetes mellitus E10.x, E11.x, E13.x, E14.x

Hypercholesterolemia E78.0

Coronary heart disease I.25.x

Peripheral vascular disease I70.x, I71.x, I73.1, I73.8, I73.9, I77.1, I79.0, I79.2, K55.1,

K55.8, K55.9, Z95.8, Z95.9

Chronic pulmonary disease I27.8, I27.9, J40.x–J47.x, J60.x–J67.x, J68.4, J70.1, J70.3

Smoking F17.2

Obesity E66.x

Chronic kidney disease N18.x, I12.0, I13.1, Z99.2

Pneumonia J13-J18, J69

Acute respiratory insufficiency J95.1, J96.0

Adult respiratory distress syndrome J80.x

Myocardial infarction I21.x

Stroke I63.x, I64.x

Postoperative Bleeding T81.0, D62, R57.1, R58

Neurologic complications G06.1, G06.2, G82.6, G95.2, G97, G97 (related to spinal puncture)

CHOP codes used to define surgical procedures

Coronary artery bypass graft Z36.1-Z36.3, Z36.9

Valve surgery Z35.1-Z35.3

Other cardiac surgery Z35.4-Z35.8, Z37.1, Z37.31, Z37.4, Z38.34, Z39.21

Extracorporeal membrane oxygenation Z39.65

Intra-aortic balloon pump Z37.61

Need for surgical revision Z39.4, Z39.98
